# Region Specific and Worldwide Distribution of Collagen-Binding M Proteins with PARF Motifs among Human Pathogenic Streptococcal Isolates

**DOI:** 10.1371/journal.pone.0030122

**Published:** 2012-01-11

**Authors:** Silvana Reißmann, Christine M. Gillen, Marcus Fulde, René Bergmann, Andreas Nerlich, Reena Rajkumari, Kootallur N. Brahmadathan, Gursharan S. Chhatwal, D. Patric Nitsche-Schmitz

**Affiliations:** 1 Department of Microbial Pathogenesis, Helmholtz Centre for Infection Research, Braunschweig, Germany; 2 Department of Clinical Microbiology, Christian Medical College, Vellore, Tamil Nadu, India; Universite Libre de Bruxelles, Belgium

## Abstract

Some of the variety of *Streptococcus pyogenes* and *Streptococcus dysgalactiae* ssp. *equisimilis* (SDSE) M proteins act as collagen-binding adhesins that facilitate acute infection. Moreover, their potential to trigger collagen autoimmunity has been implicated in the pathogenesis of acute rheumatic fever and attributed to a collagen-binding motif called PARF (peptide associated with rheumatic fever). For the first time we determine the rate of clinical isolates with collagen-binding M proteins that use a PARF motif (A/T/E)XYLXX(L/F)N in a defined geographic region, Vellore in South India. In this region both, incidence of streptococcal infections and prevalence of acute rheumatic fever are high. M proteins with PARF motif conferred collagen-binding activity to 3.9% of 153 *S. pyogenes* and 10.6% of 255 SDSE clinical isolates from Vellore. The PARF motif occurred in three *S. pyogenes* and 22 SDSE M protein types. In one of the *S. pyogenes* and five of the SDSE M proteins that contained the motif, collagen-binding was impaired, due to influences of other parts of the M protein molecule. The accumulated data on the collagen binding activity of certain M protein types allowed a reanalysis of published worldwide *emm*-typing data with the aim to estimate the rates of isolates that bind collagen via PARF. The results indicate that M proteins, which bind collagen via a PARF motif, are epidemiologically relevant in human infections, not only in Vellore. It is imperative to include the most relevant collagen-binding M types in vaccines. But when designing M protein based vaccines it should be considered that collagen binding motifs within the vaccine antigen remain potential risk factors.

## Introduction

*Streptococcus pyogenes* (group A streptococci, GAS) and *Streptococcus dysgalactiae* subsp. *equisimilis* (herein abbreviated SDSE) cause similar diseases in humans, which comprise pharyngitis, suppurative skin infections, as well as invasive infections and streptococcal toxic shock syndrome [1]–[3], the latter two causing high morbidity and mortality. Severe immune sequelae, namely poststreptococcal glomerulonephritis and acute rheumatic fever (ARF) can arise in the wake of common *S. pyogenes* and SDSE infections of the throat and skin [3]–[5]. Even though ARF has become rare in most affluent populations, it remains an important health problem in certain regions of the globe, particularly developing and newly industrializing countries [6] as well as tropical territories [7], [8].

*S. pyogenes* and the vast majority of SDSE isolates from human infections possess *emm* genes [9]–[11], which encode for a prominent pathogenic factor, the M protein. M proteins mediate adhesion to host cells and connective tissues as well as cell invasion. Moreover, these bacterial surface proteins act as antiphagocytic factors that are crucial for defense against human innate immunity (for references see [12]–[15]). High sequence variability in the N-terminal region of M proteins forms the basis of *emm* genotyping [16]. More than 200 distinct *emm* types are recorded in public databases today. In addition to other streptococcal components [17] M proteins are considered as potential vaccine antigens [18]–[21]. However, a risk of serious side effects in humans, that may be caused by molecular mimicry of coiled-coil proteins [1], [12], [22] or other features of M proteins, complicates the development of M protein based vaccines [19], [22], [23]. Some M proteins bind collagen; a function that facilitates streptococcal infections by increasing colonization of connective tissue [24]–[27]. Moreover, collagen binding by certain M proteins elicits collagen autoimmunity, which is also observed in ARF patients. This suggests collagen-binding M proteins as triggers of this disease [25], [28]. Their interaction with collagen is mediated by a **(A/T/E)**X**YL**XX**LN** amino acid sequence located in the N-terminal hypervariable region of the M protein and referred to as PARF (peptide associated with rheumatic fever) [28], [29]. Notably, the PARF motif is part of the M protein type 3 (M3), a protein that was used in a vaccination trial in which some of the vaccinees developed ARF or similar symptoms [23]. Researching the interaction between M protein and collagen promises a better understanding of its role in streptococcal diseases and as a potential risk factor to be considered in the design of M protein based vaccines. The relevance of collagen-binding M proteins as triggers of autoimmunity, in vaccine development or as virulence factors in acute infections depends on their epidemiological role worldwide and in specific geographic regions. This has motivated the following study.

## Materials and Methods

### Bacterial strains

*S. pyogenes* and SDSE isolates from human infections were collected at the Christian Medical College, Vellore. The collection comprised 153 *S. pyogenes* and 255 SDSE isolates from different suppurative foci and invasive infections. *Emm* types of 254 of the 255 SDSE strains were described previously [11]. The additional isolate was typed as stCQ343. None of isolates from Vellore was included in previous studies on collagen-binding M proteins [24], [25], [28], [29]. The *S. pyogenes* M31 strains included in binding experiments were not isolated in Vellore, but a courtesy of D. R. Johnson from the University of Minnesota Medical School.

### Sequencing of emm genes, sequence analysis, coiled-coil prediction and alignment of M proteins

*emm* genes were amplified from streptococcal genomic DNA by PCR and sequenced as described [11]. Sequence data were processed and analyzed using the software BioEdit version 7.0.1 (Isis Pharmaceuticals). “PARF motif” designates the amended (**A**/**T**/**E**)X**YL**XX(**L**/**F**)**N** consensus while “prototypical PARF motif” refers to the previously published (**A**/**T**/**E**)X**YL**XX**LN** consensus [28]. “PARF-like” denotes sequences, which are similar to PARF, but differ from the consensus in one of the conserved amino acids (positions 1, 3, 4, 7 or 8). Own sequencing data, untrimmed M protein sequences from the CDC (http://www.cdc.gov/ncidod/biotech/strep/strepblast.htm; 1180 sequences) and GenBank database [30] (648 sequences, 192 of them longer than 300 amino acids, 103 of them comprising the N-terminal signal peptidase cleavage site and the C-terminal LPSTG sequence) were analysed for the presence of PARF motifs using the search function of BioEdit for user-defined motifs (YLXXLN and YLXXFN). ClustalW was used for multiple alignments [31]. Prediction of parallel homo-dimeric coiled-coil structures in the N-terminal sequences of M proteins was performed with Paircoils2 [32] at a minimum search window length of 28.

### Collagen binding assays

Collagen binding to streptococci was tested as described previously [25]. Streptococci were grown overnight (37°C, 5% CO_2_) in Tryptic Soy Broth (TSB, Becton Dickinson), washed and resuspended in Phosphate Buffered Saline containing 0.05% Tween 20 (PBST) to a concentration of 10^8^ bacteria per ml. When necessary, 750 µl of the bacterial suspension was treated with 195 U of hyaluronidase (AppliChem) for 2.5 h at 37°C shaking at 900 rpm, then washed and resuspended in 750 µl PBST. Bacterial suspensions (250 µl) were incubated for 45 min with 30 ng (100,000 cpm) of ^125^I-labelled collagen IV (from human placenta, Sigma-Aldrich). Unbound collagen was removed by washing in 1 ml PBST before radioactivity of the pellet was quantified in a Wallac 1470 Wizard gamma counter (PerkinElmer). Data were analyzed using Microsoft Excel. Error bars indicate the standard deviation of triplicate measurements.

### Surface plasmon resonance (SPR) measurements

Recombinant M proteins stG2078, M55 and M31.5 were produced and used in SPR measurements as described previously [28]. Briefly, recombinant M proteins were injected for 2 min over immobilized human collagen IV (400 RU) at a flow rate of 60 µl/min. Data were analyzed using BiaEvaluation 3.0.

## Results

### Distribution of the PARF motif among streptococci from Vellore, India

The epidemiological relevance of streptococcal M proteins with collagen-binding PARF motif was investigated by sequencing the *emm* genes of *S. pyogenes* and SDSE strains that were isolated from human clinical infections in Vellore. The incidence of streptococcal diseases and prevalence of ARF (1.0 per 1000 school children [33]) is high in this geographic region. *Emm* types of *S. pyogenes* isolates are given in table 1. The *emm* types of 254 of 255 SDSE isolates have been reported earlier [11]. The 225^th^ was a type stCQ343 isolate. Previously identified collagen-binding M proteins with PARF motif [28], [29] occurred in 1 of the 47 *S. pyogenes emm* types (Table 1 and 2) and in 5 of the 45 *emm* types of SDSE clinical isolates [11] from Vellore (Table 2). An additional 13 distinct *emm* types, one from *S. pyogenes* and 12 from SDSE, coded for M proteins with prototypical PARF sequences, which hitherto had not been tested for collagen binding (Table 2).

**Table 1:** *Emm-t*yping of clinical *S. pyogenes* isolates from Vellore.

*emm t*ype	no. of strains
emm100	4
emm102	3
emm104	4
emm105	1
emm108	7
emm11	13
emm110	6
emm111	1
emm112	9
emm113	2
emm12	1
emm15	3
emm18	2
emm25	2
emm28	5
emm3^a^	6
emm42	2
emm44	2
emm49	6
emm53	1
emm55	2
emm56	4
emm58	1
emm60	3
emm63	7
emm66	1
emm68	1
emm69	6
emm74	2
emm77	7
emm80	3
emm82	6
emm85	2
emm88	1
emm89	1
emm92	3
emm93	4
st11014	1
st1398	8
st1731	1
st212	1
st2147	2
st2460	1
stD432	2
stKBN3	1
stKBN8	1
stNS554	1

a emm3.22 (3 isolates); emm3.23 (3 isolates).

**Table 2:** PARF motifs of *S. pyogenes* and *S. dysgalactiae* subsp. *equisimilis* isolated in Vellore.

PARF motif^a^	collagen binding^b^	M protein	No. of strains in the collection	
			*S. pyogenes*	SDSE
**A**E**YL**KA**LN**	+^c^	stG10		2
**A**E**YL**KG**LN**	+^d^	M3	3	
**A**R**YL**ET**LN**	+^c^	stC5344		1
**A**R**YL**KR**LN**	+^c^	stC-NSRT2.0		1
EA**YL**KR**LN**	+^c^	stG2574		3
TQ**YL**KR**LN**	+^c^	stC2sk		3
	+	stG97		2
**A**D**YL**KT**LN**	+	stC6746		1
**A**E**YL**KN**LN**	+	M31.5		2
**A**E**YL**KGF**N** e	+	M3.22	3	
**A**E**YL**RS**LN**	+	stG211.1		2
**A**N**YL**KE**LN**	−	stC46		1
	−	stCK401		2
**A**N**YL**KT**LN**	+	stG120.1		2
	−	stG120.0		1
	−	stGM220		1
**A**R**YL**KK**LN**	+	stG351		1
**A**T**YL**KE**LN**	−	M55	2	
**T**E**YL**KR**LN**	−	stGrobn		5
**T**E**YL**KS**LN**	+	stCQ343		1
	+	stG211.0		2
**T**K**YL**KR**LN**	+	stC922		4

a Amino acids conserved or typical for PARF are shown in bold.

b Positive strains bound more than 20% of the added collagen IV before and after hyaluronidase treatment.

c, d Collagen binding of this M protein is shown in [29] or [28], respectively.

e The motif did not fully match the previously reported consensus [28] but was positive for collagen binding.

### Collagen binding of isolates with PARF(-like) motifs

**Figure 1:**
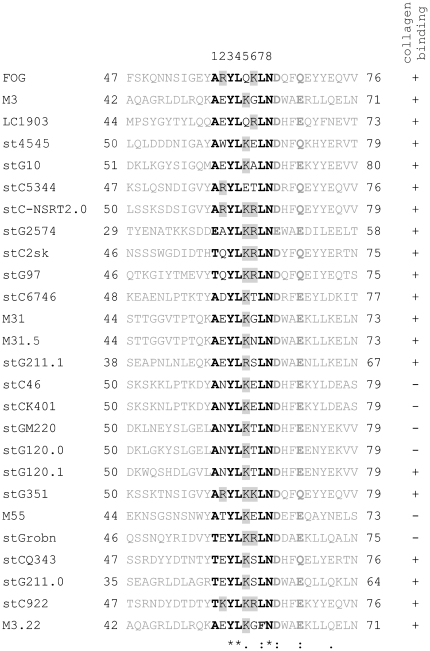
Known *S. pyogenes* and SDSE collagen-binding M proteins harboring PARF. M protein designations are given on the left. The PARF motif is shown in *black letters* and eleven flanking amino acids on both the N-terminal and C-terminal side in *grey letters*. Collagen-binding activity is indicated on the right with ‘+’ when positive or with ‘−’ when negative. Numbers flanking the sequence indicate its position within the mature protein. Amino acids conserved or typical for PARF are shown in *bold*. Identical amino acids (*), conserved substitutions (:), and semi-conserved substitutions (.) are indicated as determined by multiple alignment. Amino acids with basic side chains in positions 2, 5 or 6 are highlighted in *grey*.

**Figure 2:**
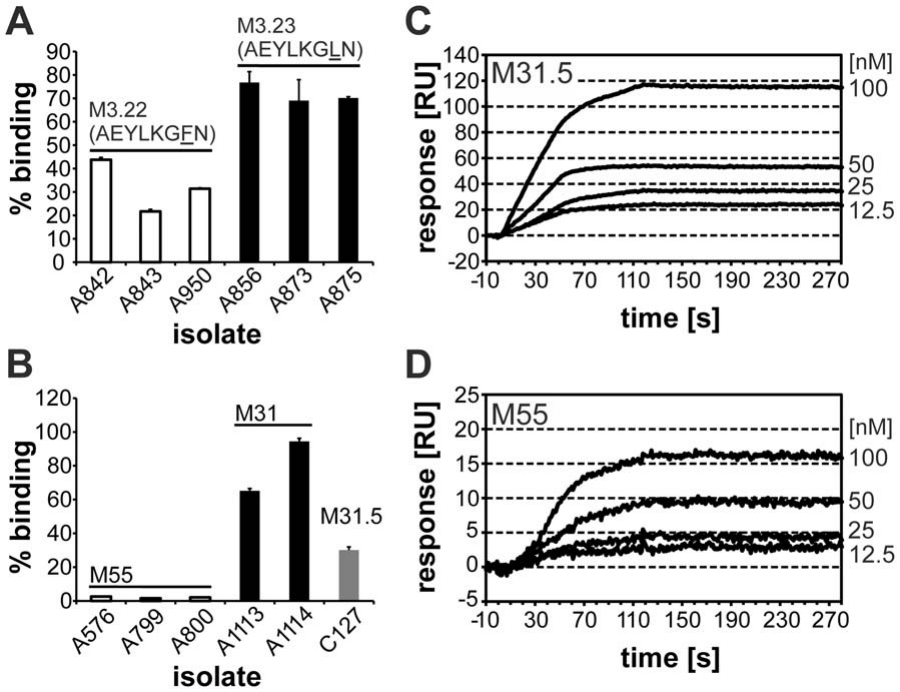
Collagen binding to M3, M31 and M55. (*A*) *S. pyogenes* strains that expressed the M protein M3.22 (*white bars*) or M3.23 (*black bars*) or (*B*) *S. pyogenes* strains that expressed M protein M55 (*white bars*) or M31 (*black bars*) and a SDSE strains that expressed M31.5 (*grey bar*) were compared for binding of collagen IV. Strain designations are indicated in the abscissa. The binding capacity was expressed as the bound percentage of added radiolabelled collagen IV. The average of three experiments is depicted with standard deviation. In (*A*) PARF sequences of the M proteins are shown above. (*C* and *D*) SPR sensograms of the interaction between immobilized human collagen IV (400 RU) and M protein M31.5 (*C*) or M55 (*D*) as analyte in the liquid phase were recorded upon injection of the analyte in concentrations that are indicated on the right in nM. Injections started at t = 0 s and ended at t = 120 s.

**Figure 3:**
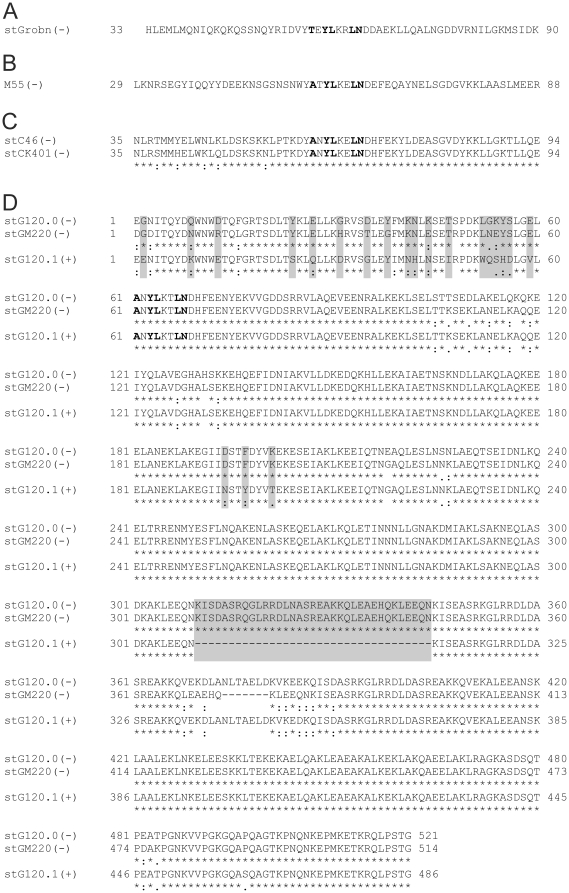
M proteins with predicted PARF motifs that did not bind collagen. Protein sequences of four types or groups of M proteins that harbored a prototypical PARF motif but had members that did not bind collagen (*A–D*). M protein designations are given on the left together with the results of collagen IV binding experiments that are given in brackets with ‘+’ for binding and ‘−’ for non-binding proteins. Numbers that flank the sequence indicate its position within the mature M protein without signal peptide. In *C* and *D* identical amino acids (*), conserved substitutions (:), and semi-conserved substitutions (.) in two or three compared sequences are marked below. Amino acids characteristic for PARF are printed in *bold*. In *D* residues that distinguish the collagen-IV binding stG120.1 from the non-binding stG120.0 and stGM220 are highlighted in *grey*.

All *S. pyogenes* isolates of the collection and representative SDSE strains with previously untested PARF-positive M proteins were examined for binding to human collagen IV (Table 2, Figure 1). Strains with M proteins, bearing PARF-like sequences were also included in the binding experiment. PARF-like sequences differ in one of the motif's conserved positions 1, 3, 4, 7 or 8 (Table 3) from the previously published [29] or amended (**A**/**T**/**E**)X**YL**XX(**L**/**F**)**N** consensus. Amendment is based on the collagen-binding activity of M3.22, shown in this study (Table 2, Figure 2*A*). As the streptococcal hyaluronic acid capsule is a potential collagen-binding factor [25], protein-dependent collagen interactions were verified in experiments with hyaluronidase treated bacteria. Several streptococcal factors including M proteins bind collagens [26], [27], [34], [35]. However, based upon a cut-off value of 20% bound ligand [29], only strains that expressed M proteins with a PARF motif bound collagen IV after hyaluronidase treatment (Table 2). The data indicated that PARF was pivotal for this interaction between M protein and collagen that confers a high binding capacity for collagen IV to the streptococci [28]. However, of the 16 newly discovered M proteins with PARF motif (14 distinct *emm* types) that were examined in this study, only ten bound collagen based upon the cut-off value (Table 2). Four types or groups of M proteins did not bind collagen IV despite harboring PARF motifs (Figure 3). The sequence **A**N**YL**KT**LN** occurred in three distinct M proteins: stGM220, stG120.0 and stG120.1 (Figure 3*D*). Interestingly, only the strain that expressed stG120.1 bound collagen IV (56% bound ligand), while strains expressing stG120.0 or stGM220 did not (6% and 14% bound ligand). Expression of the M protein by the tested strains was confirmed by examination of their typical fibrinogen interaction (not shown). Sequences of the collagen IV binding stG120.1 and of the non-binding stG120.0 and stGM220 protein were identical within the first 36 amino acids that C-terminally flank the PARF motif. Identity between stG120.1 and stGM220 extended to 125 amino acids. Lack of a C-repeat (35 amino acids) after position 309 of the mature stG120.1 protein discriminates it from the two non-binding types stGM220 and stG120.0. These two non-binding M proteins, while highly similar to one another, differed considerably from the collagen-binding M protein stG120.1 within the N-terminal region flanking the PARF motif (Figure 3*D*). Both, sequence variations either N-terminal or C-terminal from the PARF motif that distinguish the collagen binding stG120.1 from the two non-binding M proteins could govern the collagen binding activity of their PARF motif.

**Table 3:** PARF-like motifs found in M proteins of *S. pyogenes* and *S. dysgalactiae* subsp. *equisimilis* isolated in Vellore.

PARF-like motif^a^	M protein
**A**KL**L**EE**LN**	stG2078
DR**YL**DK**L**F	stG5420
**E**KN**L**EA**LN**	stG840
**E**RD**L**KD**LN**	stC6979
IN**YL**KG**LN**	stC1741
IS**YL**KD**LN**	stKNB8
LD**YL**KK**L**D	M66, M68, M92^b^, M104, M110^b^, st1389.1, st1389.2
LN**YL**KK**L**D	M110^b^
LT**YL**KK**L**D	M92^b^
PED**L**KR**LN**	stGL265.1
PED**L**KS**LN**	stGL265.0
RH**YL**KK**L**D	M28, M88, st1731
RH**YL**KQ**L**D	stNS554
RH**YL**RQ**L**D	M77, M102, M112, st212
VN**Y**VKI**LN**	stG7882

a Amino acids conserved or typical for PARF are shown in bold.

b Proteins M110 and M92 harbored two PARF-like motifs.

While M proteins of the other *emm*3 subtypes contain a prototypical PARF motif (**A**E**YL**KG**LN**), in M3.22 the leucine residue in position 7 is substituted by phenylalanine (**A**E**YL**KG**FN**). Comparison of collagen IV binding by six individual *emm*3 isolates revealed a reduced binding capacity of type *emm*3.22 as compared to *emm*3.23 streptococci that harbor the prototypical PARF motif (Figure 2*A*). Still, binding of more than 20% of the added collagen IV by *emm*3.22 streptococci, indicated that position 7 of the PARF motif tolerates substitution of leucine by phenylalanine without full loss of the collagen binding function. Hence, position 7 is more tolerant to amino acid substitution than previously known [29]. None of the 22 distinct *emm* types with PARF-like motifs (Table 3) that were tested in this study was associated with collagen IV binding. This provides further evidence for the pivotal role of the consensus sequence for this interaction. In summary the experiments demonstrate that PARF is the dominant, if not exclusive, collagen-binding motif of M proteins, that endows streptococci with a high binding capacity for collagen IV.

### Collagen binding by recombinant M proteins

Previously, M3 was the only known PARF-positive M protein of *S. pyogenes* that has been shown to bind collagen IV with high affinity [25], [28], [36]. It remained to be investigated if PARF-dependent collagen binding was limited to this single, though epidemiologically important M type [37]. Like M3, *S. pyogenes* M proteins M55 and M31 possessed a PARF motif. While M55 belongs to one of the most common *S. pyogenes emm* types isolated in Pacific Island Countries and from Indigenous Australians [37], M31 appears to be of minor epidemiological relevance worldwide [37]. The M31 protein is typically found in *S. pyogenes*, but the Vellore collection contained SDSE strains that expressed a M31.5 protein variant. Streptococci that expressed M31 or M31.5 exerted collagen-binding activity, while M55 carrying strains did not (Figure 2*B*). To examine the interaction in an additional experimental approach, collagen binding of recombinant proteins M55 and M31.5 was compared using surface plasmon resonance measurements (Figure 2*C* and *D*) in which both proteins bound to immobilized human collagen IV in a concentration dependent manner. M protein stG2078, that lacks a PARF motif, served as a control and did not produce significant responses (<4 RU). Compared to M31.5, M55 protein had a lower affinity for collagen IV as indicated by apparent dissociation constants of 0.6 nM and 5 nM, respectively. The apparent dissociation constants are based on the Langmuir model for 1∶1 interactions. However, sigmoid shapes of association curves indicated a higher complexity of the binding mechanisms. In summary, the results identified M31 protein of *S. pyogenes* and its SDSE ortholog M31.5 as collagen-binding M proteins and demonstrate a reduced collagen affinity of M55. The latter explains the lack of high capacity collagen-binding by *emm*55 *S. pyogenes* strains (Figure 2*B*).

### Role of coiled-coil domains in collagen-binding M proteins

**Figure 4:**
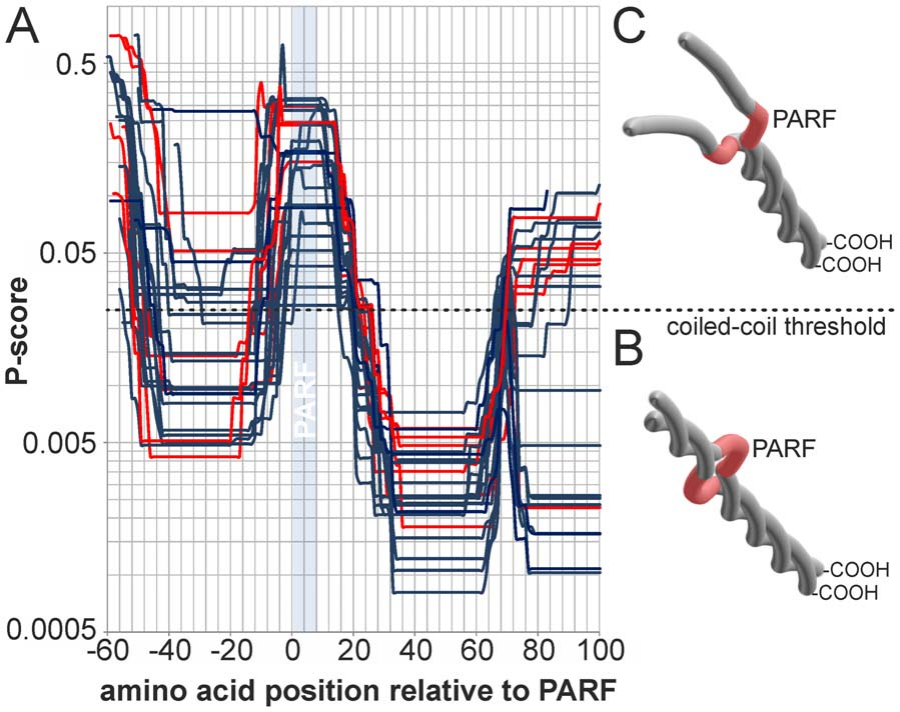
Prediction of N-terminal coiled-coil structures in PARF-positive M proteins. (*A*) Coiled-coil structure prediction for the N-terminal sequences of the PARF-positive M proteins is given as P-scores vs. the amino acid position relative from the PARF motif with the first amino acid of the motif being position 1. The figure shows the superposition of all curves without indicating protein designations, however, collagen-binding M proteins are shown in *blue* and non-binding M proteins are shown in *red*. Single curves for all M proteins are provided as a supplementary figure (Figure S1). The position of the PARF motif is highlighted in *light blue*. A *black dashed line* indicates the threshold value for coiled-coil prediction. Values below 0.025 indicate a coiled-coil structure. The prediction separates the M proteins into two classes; one class with (*B*) and one without (*C*) an N-terminal coiled-coil domain. They are shown in schematic representations that are not drawn to scale. *B* and *C* are not based on structural data other than coiled-coil prediction. The PARF motif is highlighted in *red*. The C-terminal end is labelled (-COOH).

**Figure 5:**
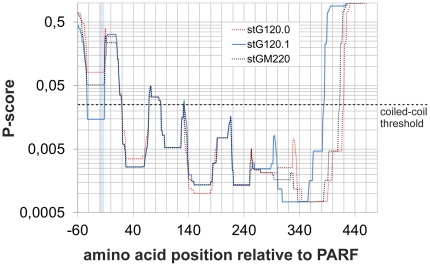
Prediction of coiled-coil structures in PARF-positive M proteins. Coiled-coil structure prediction for the N-terminal sequences of the PARF-positive M proteins stG120.0 (*red dotted line*), stG120.1 (*light blue solid line*) and stGM220 (*black dotted line*) is given as P-scores vs. the amino acid position relative from the PARF motif with the first amino acid of the motif being position 1. Isolates with M protein stG120.1 bind collagen IV, while strains that carry one of the other two M proteins do not. The position of the PARF motif is highlighted in *light blue*. A *black dashed line* indicates the threshold value for coiled-coil prediction. Values below 0.025 indicate a coiled-coil structure.

M proteins form coiled-coil dimers [24], [38]–[41]. Such a ternary structure of the molecule may impact folding, accessibility and thus the collagen binding function of the PARF motif. Therefore, the position of the PARF motif was determined in relation to predicted coiled-coil domains (Figure 4*A*). A high probability for coiled-coil formation was observed in close C-terminal proximity to the PARF motif in all examined M proteins. In contrast, the PARF motifs themselves form a sequence section with minimal coiled-coil probability. Such interruption of the coiled-coil structure may be a prerequisite for collagen binding. Influences of the N-terminal M protein sequence on PARF activity may depend on the ability of the N-terminal end to form a parallel inter-chain coiled-coil domain (Figure 4*B*). The predictions separated M proteins with and without an N-terminal coiled-coil, but did not discern collagen-binding M proteins from inactive ones (Figure 4*B* and *C*). Thus, the structural influences that govern the collagen-binding activity of the PARF motif are more complex in nature and seem independent of the M protein's ability to form an N-terminal inter-chain coiled-coil structure. Comparing coiled-coil predictions for the full length M proteins (Figure 5) suggested that the most drastic structural differences between the collagen IV binding type stG120.1 and the non-binding types stG120.0 and stGM220 lie N-terminal from the PARF motif. C-terminal from the PARF motif, the sequences show a very similar coiled-coil prediction over a stretch of 240 amino acids. Because of the lacking C-repeat in stG120.1 (Figure 3D) coiled-coil-prediction of the M proteins 164 most C-terminal residues is shifted relative to stG120.0, but shows a similar curve shape. The coiled-coil prediction analysis indicates that major structural differences, which discern stG120.1 from the non-binding M proteins, are limited to the N-terminal flanking region of PARF and to a distant C-terminal part of the sequence. Since both could be the cause of impaired collagen binding, the origin of inactivating influences remains to be identified.

### Global and regiospecific distribution of streptococci with collagen-binding M proteins

The presented data show that a PARF motif is not a sufficient but a necessary condition for high affinity binding of collagen IV by streptococcal M proteins. In this study, combining PARF-based prediction with experimental validation of collagen IV binding activity disclosed that 3.9% of 153 *S. pyogenes* and 10.6% of 255 SDSE clinical isolates from Vellore possessed a collagen-binding M protein with PARF. Taking previous studies into account a large number of binding M types is now identified (Figure 1). Searching the M protein database of the Centers for Disease Control and Prevention (http://www.cdc.gov/ncidod/biotech/strep/strepblast.htm) revealed an additional 14 M proteins with PARF motif that remain to be tested for collagen binding (Table 4). However, the most frequent PARF-positive M types worldwide [37] and all except one M type with PARF motif that are reported in the herein reanalyzed studies [8], [42]–[47] are now tested for PARF-dependent collagen interaction. This in turn allowed the following estimations on global and region specific distributions of collagen-binding M proteins with PARF motif. Worldwide, due to a low epidemiological relevance of *emm*31, stKBN7, stM3, stPILA, st0726, st1692, st3720, st3857 strains in the past [37], the rate of *S. pyogenes* strains that possess collagen-binding M proteins with PARF motif was largely determined by *emm*3 strains. Based on the data of Steer and colleagues [37] it can be estimated that worldwide about 7% of the *S. pyogenes* strains possessed a collagen-binding M protein with PARF. In different geographic regions the estimated rate of *S. pyogenes* strains with active PARF motif ranged from less than 2% in Asia over 3.5% in Africa and 7.5% in the established market countries of Europe, North America, Australia, Japan and Hong Kong to more than 10% in the Middle East. A rate of 26.5% *emm*3 strains in pediatric patients in China that was documented for the years 1993–1994 and a subsequent drastic decrease until 2005–2006 documents the rate's potential for high temporal variability [42]. PARF motifs conferred collagen binding activity to a variety of distinct M proteins of SDSE (Table 2, Figure 1). Analysis of studies on SDSE, which were not limited to invasive cases of infection, revealed rates of 4.4%, 4.7% and 13.8% in Thailand [43], Norway [44] and Portugal [45], respectively, for isolates with PARF-positive collagen-binding M proteins. For a study from Japan [46] the estimated rate ranged between 13.8% and 14.5% since collagen binding activity of stG4974 remains untested (Table 4). Based on community surveillances, collagen-binding M proteins with PARF motif occurred in 8.7% of the strains from Mumbai school children [47] and in 5.9% of the strains isolated from Indigenous Australians [8]. Collectively, this retrospective analysis of *emm* typing data indicates that collagen-binding M proteins with PARF motif are frequently involved in clinical cases of *S. pyogenes* and SDSE infection both, in Vellore and on a global scale.

**Table 4:** Untested additional M proteins with PARF-like motifs found in the CDC and GenBank database.

Species	PARF motif^a^	M protein
*S. pyogenes*	**A**E**YL**KG**LN**	st0775
		st1692
		st3720
		stM3
		st4050
	**A**D**YL**KK**LN**	stPILA
	**T**K**YL**KE**LN**	stKNB7
		
SDSE	**A**Q**YL**KK**LN**	stCN204
	**T**Q**YL**KS**LN**	stG354
	**A**Q**YL**RE**LN**	stG1750
	**A**E**YL**QR**LN**	stG4974
	**A**R**YL**RE**LN**	stG5063
	**T**N**YL**KT**LN**	stGLP3
	**A**D**YL**YR**LN**	stGM203

a Amino acids conserved or typical for PARF are shown in bold.

## Discussion

*S. pyogenes* is an exclusively human pathogenic bacterium that exerts host specific interactions during infection. Animal models simulate its pathogenesis only partially, particularly the complex processes underlying streptococcal sequelae. As a consequence, deeper investigations on the role of collagen-binding M proteins in streptococcal clinical infections and sequelae require additional approaches, comprising the analysis of clinical data and causative strains collected from human patients. These clinical approaches benefit from comprehensive knowledge about the circulating streptococcal strains expressing collagen-binding M proteins. Presence of a prototypical PARF motif **(A/T/E)**X**YL**XX**LN** in the variable N-terminal amino acid sequence of M protein was suggested as predictive of a collagen binding function [28], [29]. Screening of the complete *S. pyogenes* collection in this study demonstrated that the occurrence of a PARF motif **(A/T/E)**X**YL**XX(**L**/**F**)**N** in the M protein was not a sufficient but a necessary condition for its collagen-binding activity (Figure 1) that comprises high affinity binding to collagen IV. Supportive to earlier results [28], [29], the positions 2, 5, and 6 of the PARF motifs were preferentially occupied by charged amino acids, most often with basic side chains (Figure 1). Collagen binding of M proteins stGrobn, stCQ343, stG211.0, stG97 and stC922 provided further experimental evidence for the tolerance of threonine in position 1 of the PARF motif (Figure 1). Moreover, a certain tolerance of position 7 for phenylalanine substitution was observed (Figure 2*A*), which led to the amended PARF consensus described above. Due to potential interference of other parts of the M protein with the collagen-binding motif, conclusions on the effect of isoleucine substitution in position 1 were not possible (Table 3). Conspicuously, the first amino acid following the PARF consensus was conserved (Figure 1). This suggests that the acidic side chains of aspartic or glutamic acid may contribute to the collagen-binding activity of the M proteins. In earlier experiments with synthetic peptides substitution of the aspartic acid by alanine had no influence on the collagen interaction [28]. Therefore, it is more likely that the acidic residue is important for the fold of the binding motif within the M protein rather than being a direct contact site for the collagen molecule. The fourth position following the collagen-binding motif was occupied by either glutamine or glutamic acid; amino acids of different character but similar dimensions. Contributions of additional amino acids to the collagen-binding motif of M proteins appear likely and deserve further investigation.

The epidemiological significance of collagen-binding M proteins is demonstrated by the presented data. Regionally and temporally the rates of collagen-binding M proteins that use a PARF motif differed considerably, exceeding 10% for both, *S. pyogenes* or SDSE in several studies [37], [42], [45], [46]. Previous [25], [28], [29] and herein presented experimental data on more than 100 different M protein types locate all known collagen-binding PARF motifs within the first 70 amino acids of the mature M protein. Searches in the CDC and GenBank database disclosed 14 more M proteins with PARF sequences (Table 4), but none of the PARF motifs was located more C-terminally than amino acid number 70. The GenBank database included 103 full-length sequences of mature M proteins, representing a great variety of C-terminal M protein sequences. Still, due to limited C-terminal sequence information particularly in the CDC database and due to the presumable existence of unknown M types, it cannot be excluded that some PARF-positive M proteins remain unidentified. Therefore, the herein presented reexamination of previous *emm*-typing studies, delivered estimated rates of collagen binding M proteins with PARF motif, which may be associated with some, though reasonable, uncertainty.

The presented work is limited to investigations on the PARF-dependent M protein interaction that endows streptococci with a high binding capacity for collagen including collagen IV. Nevertheless, it needs to be mentioned that other streptococcal collagen-binding factors exist [25]–[27], [34]; among them M1 protein, that does not have a PARF motif but binds collagen I and VI with high and collagen IV with low affinity. Interestingly, M1 binds to the non-collagenous moiety of collagen VI [26], [27]. The observed differences in binding specificities between PARF-positive and PARF-negative collagen binding M proteins suggest that they have dissimilar functions during infection. Even so, the epidemiological relevance of collagen-binding M proteins in human infections appears to be higher than suggested by herein presented data on PARF and requires further examination.

Rapidly progressing invasive infections and high incidence of sequelae under poor socioeconomic conditions nurture the need for a vaccine against these bacteria. Several M protein and non-M protein vaccines are in development [17]–[21]. Due to the obvious epidemiological relevance of certain PARF-positive *emm* types like *emm*3 [37] it is imperative to include them in an efficacious streptococcal vaccine. Although suspected to induce the diseases it should prevent [1], [21], [23], the M protein remains under consideration as a vaccine candidate [18]–[21]. One strategy to prevent the hazardous side effects of this antigen is to exclude or inactivate the pathogenic motifs of the M protein while preserving the epitopes that induce protective immune responses [19], [21]. This requires a profound knowledge about all confirmed or potentially autoimmunogenic motifs, including the collagen-binding site. PARF motifs reside in the N-terminal portions of M proteins that induce type-specific protective immunity and that are considered as promising vaccine antigens [19]–[21], [48]. With respect to the potential to induce autoimmunity it seems advisable to inactivate the PARF motifs in vaccine antigens, preferably with minimal changes in protein sequence and structure.

The existence of PARF-bearing M proteins that are inactive regarding collagen binding is a novel observation and a paradigm for the limits of purely sequence based functional predictions. However, their identification creates an opportunity to study the structural requirements for PARF activity and for its inactivation. Comparing amino acid sequences and coiled-coil structure predictions of PARF-bearing M proteins suggests that N-terminal sequences flanking the PARF motif or more distant C-terminal sequence variations can exert structural influences that interfere with collagen binding. The most drastic differences up to the ternary structure of these M proteins lie N-terminally from the PARF motif, but no general correlation between prediction of an N-terminal coiled-coil domain and collagen-binding activity was observed, indicating that other perhaps more complex or even individual structural influences act on the PARF motif. Therefore, a study to unveil the structural biological details of the interaction between collagen and M proteins needs a careful design; an effort that could be rewarded with therapeutic substances that interfere with streptococcal colonization, with a better understanding of autoimmunity induced by streptococci and with safe antigens for vaccines against these pathogens.

## Supporting Information

Figure S1 **Prediction of coiled-coil structures in PARF-positive M proteins.** Coiled-coil structure prediction for the N-terminal sequences of the indicated PARF-positive M proteins is depicted as P-scores vs. the amino acid position relative from the PARF motif with the first amino acid of the motif being position 1. Scores below 0.025 indicate a coiled-coil structure. The position of the PARF motif is highlighted in *light grey*.
(PDF)
