# Supplementary material for: Region Specific and Worldwide Distribution of Collagen-Binding M Proteins with PARF Motifs among Human Pathogenic Streptococcal Isolates
Source: PLoS One. 2012 Jan 11;7(1):e30122. doi: 10.1371/journal.pone.0030122 (PMC3256231; doi:10.1371/journal.pone.0030122)
Supplement: Figure S1 — Prediction of coiled-coil structures in PARF-positive M proteins. Coiled-coil structure prediction for the N-terminal sequences of the indicated PARF-positive M proteins is depicted as P-scores vs. the amino acid position relative from the PARF motif with the first amino acid of the motif being position 1. Scores below 0.025 indicate a coiled-coil structure. The position of the PARF motif is highlighted in light grey. (PDF) [file pone.0030122.s001.pdf]

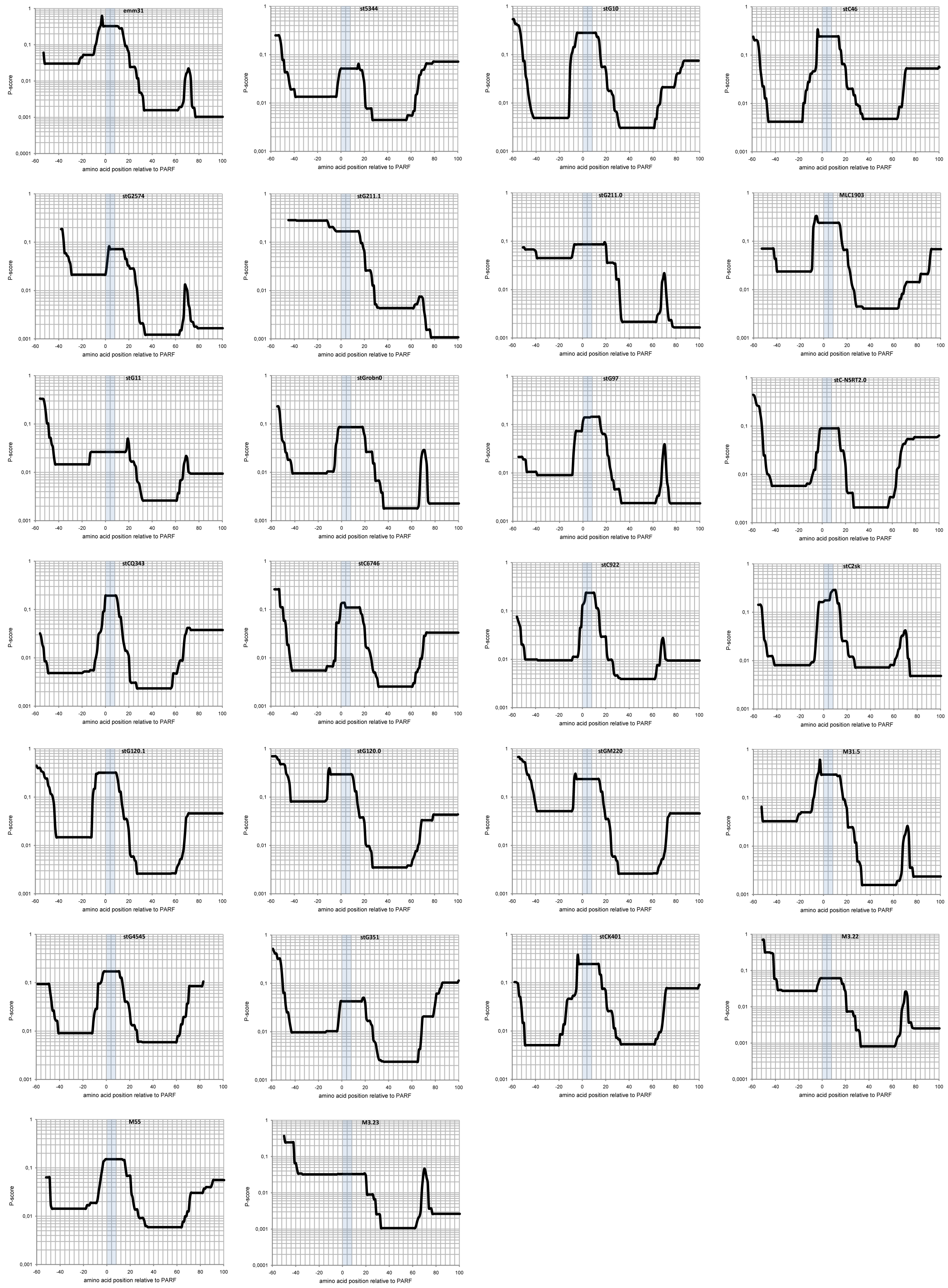

Supplementary Figure 1: Prediction of coiled coil structures in PARF-positive M proteins. Coiled coil structure prediction for the N-terminal sequences of the indicated PARF-positive M proteins is depicted as P-scores vs. the amino acid position relative from the PARF motif with the first amino acid of the motif being position 1. Scores below 0.025 indicate a coiled coil structure. The position of the PARF motif is highlighted in *light gray*.
